# Modulated DISP3/PTCHD2 expression influences neural stem cell fate decisions

**DOI:** 10.1038/srep41597

**Published:** 2017-01-30

**Authors:** Jana Konířová, Jana Oltová, Alicia Corlett, Justyna Kopycińska, Michal Kolář, Petr Bartůněk, Martina Zíková

**Affiliations:** 1 Institute of Molecular Genetics AS CR v.v.i., Vídeňská 1083, 142 20 Prague 4, Czech Republic

## Abstract

Neural stem cells (NSCs) are defined by their dual ability to self-renew through mitotic cell division or differentiate into the varied neural cell types of the CNS. DISP3/PTCHD2 is a sterol-sensing domain-containing protein, highly expressed in neural tissues, whose expression is regulated by thyroid hormone. In the present study, we used a mouse NSC line to investigate what effect DISP3 may have on the self-renewal and/or differentiation potential of the cells. We demonstrated that NSC differentiation triggered significant reduction in DISP3 expression in the resulting astrocytes, neurons and oligodendrocytes. Moreover, when DISP3 expression was disrupted, the NSC “stemness” was suppressed, leading to a larger population of cells undergoing spontaneous neuronal differentiation. Conversely, overexpression of DISP3 resulted in increased NSC proliferation. When NSCs were cultured under differentiation conditions, we observed that the lack of DISP3 augmented the number of NSCs differentiating into each of the neural cell lineages and that neuronal morphology was altered. In contrast, DISP3 overexpression resulted in impaired cell differentiation. Taken together, our findings imply that DISP3 may help dictate the NSC cell fate to either undergo self-renewal or switch to the terminal differentiation cell program.

Neural stem cells (NSCs) are defined by their ability to self-renew through mitotic cell division and to differentiate into the various neural cell types: neurons, astrocytes and oligodendrocytes^1^,^2^. In the developing brain, NSCs first undergo symmetric self-renewal to expand the stem cell pool, which is followed by asymmetric neurogenic and gliogenic cell division to generate neurons and glia, respectively^3^. In the adult brain, NSCs reside in niches with specific molecular and cellular characteristics and whose specification is regulated by a large number of factors in each niche. Transduction of extracellular niche signals triggers a signaling cascade that activates intracellular regulatory mechanisms, including transcription factors, epigenetics and metabolism that control cell proliferation and differentiation (reviewed in ref. 4).

NSCs isolated from fetal^5^,^6^,^7^ and adult^8^,^9^,^10^,^11^ mammalian central nervous systems have previously been propagated *in vitro* in the presence of epidermal growth factor (EGF) and fibroblast growth factor 2 (FGF-2) to generate multicellular aggregates called neurospheres^6^,^11^,^12^. An alternative method of producing NSCs *in vitro* is via embryonic stem (ES) cells^13^,^14^,^15^. To date, neural differentiation of ES cells has been achieved using several published protocols that include treating ES cell aggregates with retinoic acid^16^ or co-culturing ES cells on monolayers of bone marrow-derived stromal PA-6 cells^17^. Interestingly, recent studies have revealed that neither multicellular aggregation nor co-culture is necessary for ES cell neural commitment. Instead, eliminating signals that trigger alternative cell fates and the presence of EGF and FGF-2 are sufficient for ES cells to develop into neural precursors^15^.

The NS-5 cell line represents NSCs derived from mouse ES cells. Differentiation of ES cells into neural precursors was induced in monolayer; lineage selection for cells expressing pan-neural gene *Sox1* was used to eliminate NSCs from undifferentiated ES cells and from non-neural differentiated cells. Subsequent cultivation of cells in the presence of EGF and FGF-2 resulted in a homogenous population of adherent bipolar cells that can be continuously symmetrically expanded in adherent monoculture without any spontaneous differentiation. Moreover, NS-5 cells represent tripotent NSCs, so even after prolonged expansion, they are still capable of generating neurons, astrocytes and oligodendrocytes under particular conditions *in vitro*^18^,^19^.

The Dispatched 3 gene (*Disp3*), also known as *Ptchd2* or *KIAA1337*, encodes a 13-transmembrane domain-containing protein, highly expressed in neural tissue and regulated both *in vivo* and *in vitro* by thyroid hormone^20^. Previously, ectopic expression of DISP3 in multipotent cerebellar progenitor cells was shown to promote cell proliferation and modulate expression of the genes involved in tumorigenesis. Further investigation revealed that *DISP3* mRNA levels are significantly elevated in the human brain cancer medulloblastoma^21^.

Sequence alignments with structurally related proteins (HMGCR, SCAP, NPC1, NPC1L1, 7DHCR, PTCH1, PTCH2, DISP1 and DISP2) have shown that DISP3 contains a putative sterol-sensing domain (SSD). Functional analysis of these SSD-containing proteins revealed a link between the SSD and cholesterol homeostasis or cholesterol-linked signaling^22^.

Lipid metabolism is fundamental for the brain development, but deciphering its role under normal and pathological conditions is difficult due to the brain lipid content complexity. Under normal conditions, neurogenesis requires brain fatty acid synthesis^23^ and moreover, the proliferation capacity of NSCs depends on fatty acid oxidation^24^. In the pathological conditions, the accumulation of lipids is often a hallmark of affected neurogenesis. It was found that triple-transgenic Alzheimer’s disease mice accumulate neutral lipids within the subventricular zone niche, which is sufficient to inhibit NSC proliferation^25^.

In the current study we have investigated whether the levels of DISP3 expression could affect the self-renewal and/or differentiation potential of NSCs. Given that DISP3 expression is elevated in medulloblastoma and that distinct molecular subtypes of medulloblastoma can be characterized by specific neural stem cell molecular signatures^26^, we wished to elucidate what role DISP3 may play in the neural stem cell development.

## Materials and Methods

### Cell culture and differentiation

NS-5 cells were a generous gift from Dietman Spengler (Max-Planck Institute of Psychiatry, Munich, Germany) with the permission of Austin Smith (Wellcome Trust Centre for Stem Cell Research, University of Cambridge, Cambridge, United Kingdom). Cells were cultured in a growth medium prepared by combining DMEM/F12 medium (Sigma) containing N2 supplement (Gibco) and Neurobasal medium (Gibco) containing B27 supplement (Gibco) and 2 mM L-glutamine (Gibco). The final medium was supplemented with BSA (25 μg/ml, Gibco), insulin (12.5 μg/ml, Sigma), apo-transferrin (50 μg/ml, Sigma), FGF-2 (10 ng/ml, R&D Systems) and EGF (10 ng/ml, R&D Systems). Cells were passaged every other day using 0.025% trypsin/EDTA and reseeded onto gelatine-coated dishes at a density of 1.5 × 10^4^/cm^2^. For NSC differentiation experiments, cells were seeded onto poly-ornithine/laminin-coated dishes at a density of 0.5 × 10^4^/cm^2^ and grown in standard growth medium. After 24 h in culture, the growth medium was changed to allow differentiation. To induce astrocyte differentiation, cells were incubated for four days in the standard growth medium supplemented with 1% FCS without EGF and FGF-2. For neuronal and oligodendrocyte differentiation, cells were cultured in DMEM/F12 medium (Sigma) supplemented with N2 (Gibco), FGF-2 (10 ng/ml, R&D Systems), PDGF-AA (10 ng/ml, R&D Systems) and forskolin (10 μM, Sigma). After four days, the medium was changed to DMEM/F12 medium (Sigma) supplemented with N2 (Gibco), FGF-2 (7.5 ng/ml, R&D Systems), ascorbic acid (200 μM, Sigma) and T3 (30 ng/ml, Sigma). Starting from day 5, the amount of FGF-2 in the medium was gradually reduced by removing half of the medium and replacing it with fresh medium lacking FGF-2.

### RNA preparation and real-time qRT-PCR

Total RNA was extracted from the cultured cells using a PureLink RNA Mini Kit (Ambion) according to the manufacturer’s protocol. For real-time RT-PCR, 200 ng of total RNA was reverse transcribed using random hexamer primers (Invitrogen) and M-MLV Reverse Transcriptase (Promega). cDNAs were amplified by the LightCycler^®^ 480 system (Roche) using the SYBR Green I Master mix (Roche). All reactions were run in triplicates; all mRNAs levels were normalized to *Ubb* mRNA.

### Primers

The following primers were used: Ubb 5′-ATGTGAAGGCCAAGATCCAG-3′ and 5′-TAATAGCCACCCCTCAGACG-3′, Disp3 5′-CAGCAGCTTTGACCTCTTCA-3′ and 5′-GCAACATCTGCAGGAAGGA-3′, T7EI-Disp3 sgRNA#1 5′-GCGAATCGAGCTCATCTTTCTGG-3′ and 5′-GGAGGATGGAATAAACCCCTTT-3′, T7EI-Disp3 sgRNA#2 5′-TGTGTGATGTGTCGTCCGTA-3′ and 5′-TCACATGCGACTACACTGCT-3′, βIII-tubulin 5′-TGGACAGTGTTCGGTCTGG-3′ and 5′-CCCTCCGTATAGTGCCCTTTG-3′, GFAP 5′-TGAGGCAGAAGCTCCAAGA-3′ and 5′-CCAGGGTGGCTTCATCTGC-3′, Plp 5′-GTTCCAGAGGCCAACATCAAGC-3′ and 5′-AGCCATACAACAGTCAGGGCATAG-3′, nestin 5′-AGGCTGAGAACTCTCGCTTGC-3′ and 5′-GGTGCTGGTCCTCTGGTATCC-3′, Igfbp7 5′-TGGTGACCGGGAAAATCTGG-3′ and 5′-TGCGTGGCACTCATACTCTC-3′, Lipt1 5′-TCCACGTGGGTTGATTGAGT-3′ and 5′-GCTGCTGGGACCTTGTGCTG-3′, Dgka 5′-GCTCTGTGTCTCTAGACGAG-3′ and 5′-TGGTGAATCTCTTGGGTCTCC-3′, Brsk1 5′-CCCGAGAAAAGGCTCAGTC-3′ and 5′-GGCTACGCATGGCTACTCTG-3′, Edg8 5′-GGACCGCTGTTTCTCTTGC-3′ and 5′-GATGGGATTCAGCAGCGAGT-3′.

### Immunofluorescence and imaging

To detect proteins via immunofluorescence, cells were grown on glass coverslips, fixed in 4% paraformaldehyde, permeabilized with 0.1% Triton X-100 and blocked in a mixture of 10% NGS (Jackson ImmunoResearch) and 5% BSA (Sigma) before incubation with a primary antibody. Antibodies used included polyclonal anti-DISP3 antibody^21^ (1:500), mouse anti-βIII-tubulin (1:1000, R&D Systems), mouse anti-GFAP (1:400, Sigma), mouse anti-oligodendrocyte marker O4 (1:500, R&D Systems). DISP3 staining was visualized by the biotin-streptavidin method using streptavidin conjugated to Alexa Fluor 555 (Invitrogen), βIII-tubulin and GFAP staining by goat anti-mouse IgG Alexa Fluor 488 secondary antibody (Invitrogen) and O4 staining by goat anti-mouse IgM Alexa Fluor 555 secondary antibody (Invitrogen). To visualize nuclei, cells were stained with DAPI (Sigma). Images for automated image analysis were taken with an Operetta HTS imaging system (PerkinElmer) at 20x magnification using Harmony software. A minimum of 50 fields per slide were analyzed using the Columbus image analysis software (PerkinElmer). The number of βIII-tubulin-stained neurites was evaluated using the neurite finding tool (CSIRO Neurite Analysis method)^27^ in the Columbus software and plotted as the number of neurites in the field divided by the total number of cells in the same field. In differentiated neuronal culture, highly βIII-tubulin-positive cells with extending neurites and dense cytoplasm were considered as neurons. Neurons and primary neurites were tagged and counted manually using the ImageJ software (Cell Counter plugin). Multiple fields (20x magnification, approximately about 300 cells) per each of the cell culture types in each of the seven independent experiments were counted. Representative pictures were acquired using a Leica DMI4000B microscope.

### Virus production, transduction and transfection of NS-5 cells

The CRISPR/Cas9 method was utilized to knock-out the *Disp3* gene in NS-5 cells. The E-CRISP tool (http://www.e-crisp.org/E-CRISP/) was used to find suitable targets for mutagenesis within the *Disp3* gene. Two sites were chosen and oligonucleotides were ordered. These were then annealed and cloned into the lentiCRISPRv1 vector (Addgene # 49535) as previously described by Shalem *et al*.^28^.

Lentiviruses were prepared by co-transfecting HEK293FT cells (Thermo Fisher Scientific, R70007) with the pLentiCRISPRv1 expression vector, psPAX2 and pVSV-G plasmids using the Lipofectamine reagent (Invitrogen). Twenty-four hours post transfection viral supernatant was harvested and the virions precipitated using PEG/it (SBI). Precipitated virions were resuspended in PBS and then incubated for 24 h with NS-5 cells. Cell medium was then changed and puromycin was added 24 h later.

To generate DISP3-overexpressing NS-5 cells, the full-length human *DISP3* (GenBank AB037758.1) was cloned into the retroviral vector pBABE-Hygro. Retroviruses were prepared by transfecting the expression vector into Phoenix-ECO cells (ATCC CRL-3214) using the Lipofectamine reagent. Viral supernatant was harvested at 36 h post transfection. NS-5 cells were incubated with virus supernatant and polybrene (4 μg/ml) for 8 h before changing the medium. Hygromycin was added 48 h later.

To generate short hairpin RNAs (shRNAs) against the *Disp3* gene (exon 2, target sequences: 5′-AUCGAGCUCAUCUUUCUGG-3′, 5′-GUUCUCUCAUGACUUACUU-3′), we utilized the pSilencer Expression Vector Insert Design Tool (Ambion). The oligonucleotides for shRNA expression were manufactured by Ambion, annealed and ligated into the pSilencer 2.1-U6 puro vector (Ambion). A negative control plasmid, pSilencer 2.1-U6 puro-NC, was supplied by Ambion. NS-5 cells were transfected using the Lipofectamine reagent (Invitrogen) and puromycin was added 24 h later.

### T7 endonuclease assays

Genomic DNA was isolated using a QIAamp DNA Mini Kit (Qiagen). The genomic regions surrounding the target sites #1 (Exon 2) and target sites #2 (Exon 7) were PCR amplified using Q5 High-Fidelity DNA Polymerase (New England BioLabs) and primers T7EI-Disp3 sgRNA#1 and T7EI-Disp3 sgRNA#2. A total of 200 ng of the purified PCR product was denatured and slowly reannealed to facilitate heteroduplex formation: 95 °C for 10 min, 95-85 °C temperature ramping −2 °C/s, 85-25 °C temperature ramping −0.1 °C/s and 25 °C for 5 min. The reannealed amplicon was digested with T7 endonuclease I (New England BioLabs) at 37 °C for 20 min and analyzed in 2% agarose gel.

### Immunoblotting

Protein samples were separated by SDS-PAGE using 4–15% precast gradient polyacrylamide gels (Biorad). Proteins were transferred onto nitrocellulose membranes which were subsequently blocked in a solution containing 5% non-fat milk dissolved in TBS and 0.05% Tween-20 (TBST). Filters were incubated in primary antibody (anti-DISP3 antibody, 1:500; anti-actin, 1:500, Sigma) diluted in 1% milk/TBST overnight and then washed and incubated with secondary antibody (ECL kit, GE Life Sciences).

### Proliferation assays

Cell proliferation was measured using thymidine incorporation assays. Cells were plated at a density of 4 × 10^3^ cells/well in 96-well plates. After 12 hours, cells were pulsed with [methyl-^3^H] thymidine (UJV Rez) and cultured for an additional 8 hours. Cells were harvested onto a Filtermat using a FilterMate Harvester. Incorporated radioactivity was quantified using a MicroBeta2 Microplate scintillation counter (PerkinElmer). A cumulative growth curve was established by reseeding cells at a density of 1.5 × 10^4^ /cm^2^ every other day and counting cell numbers using a CASY Cell Counter and Analyzer System (Roche).

### Cell cycle analysis

For cell cycle analysis, cells were trypsinized (0.025% trypsin/EDTA), collected and fixed in a solution of 70% ethanol. After incubation at −20 °C overnight the cells were placed in a staining solution containing 0.1% Triton X-100 in PBS, propidium iodide (20 μg/ml, Sigma) and RNase A (200 μg/ml) for 15 min. Samples were analyzed using a BD LSRII flow cytometer and Watson pragmatic univariate analysis^29^ tool in software FlowJo (FlowJo LLC).

### Microarray analysis

RNA quality and concentration was measured using a NanoDrop 2000 spectrophotometer (Thermo Fisher Scientific) and RNA integrity was analyzed by Agilent Bioanalyzer 2100 (Agilent). Illumina MouseRef-WG-6 v2 Expression BeadChips (Illumina) were used for microarray analysis according to the manufacturer’s protocol. In brief, 250 ng of RNA was amplified using the Illumina TotalPrep RNA Amplification Kit (Ambion). The labeled RNA (1500 ng) was then hybridized onto the chip according to the manufacturer’s instructions. The analysis was performed in three to four biological replicates per group. The raw data was preprocessed using GenomeStudio software (version 1.9.0.24624; Illumina) and analyzed within the limma package^30^ of Bioconductor^31^ as described elsewhere^32^. In short, we used the normal-exponential model to remove the background noise, quantile normalization to standardize the data and log-2 transform to stabilize variance of the data. A moderated t-test of limma was used to detect transcripts differentially expressed between the Disp3 sgRNA-treated samples and controls. As the analysis was considered exploratory, we used weak criteria to select candidate genes that were differentially transcribed (Storey’s q < 0.9^33^), which corresponds to p < 0.001, and |log2FC| > 0.4. The MIAME (Minimum Information About a Microarray Experiment) compliant transcription data was deposited in the ArrayExpress database (E-MTAB-4485).

### Statistical analysis

The results obtained in the qRT-PCR analysis, proliferation measurement and automated microscopy image analysis were processed using GraphPad Prism software version 6. In the plots generated, each data point represents a biological replicate and the mean value is shown. Error bars specify standard deviation of the data. To determine the statistical significance of the results, an unpaired t-test with Welch’s correction was used. Statistical significance is denoted by asterisks, *P < 0.05, **P < 0.01, ***P < 0.001, and ****P < 0.0001.

## Results

### DISP3 expression is reduced in differentiated neural stem cells

**Figure 1:**
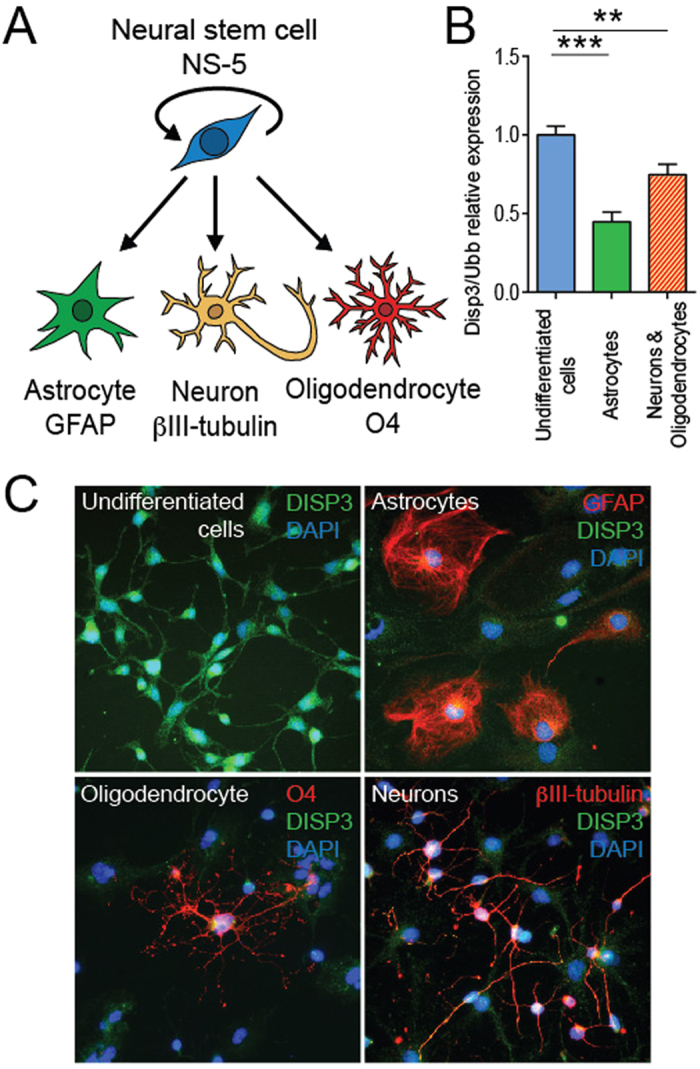
DISP3 expression alters during neural differentiation in NS-5 cells. (**A**) Multipotent neural stem cells NS-5 give rise to astrocytes, neurons and oligodendrocytes. Cell type-specific markers GFAP (astrocytes), βIII-tubulin (neurons) and O4 (oligodendrocytes) were analyzed. (**B**) Quantitative RT-PCR analysis of *Disp3* mRNA in undifferentiated and differentiated cells. *Ubb* was used as a reference gene. Bars represent the mean of three independent samples with error bars indicating standard deviation and the level of statistical significance (**P < 0.01, ***P < 0.001). Mean values: undifferentiated cells = 1.00, astrocytes = 0.45, neurons and oligodendrocytes = 0.75. (**C**) Immunofluorescence images of undifferentiated and differentiated cells stained with a polyclonal DISP3 antibody (green) and cell type-specific markers (red). DAPI (blue) was used to stain the nuclei.

NS-5 mouse neural stem cells are capable of differentiating *in vitro* into the three neural cell types: astrocytes, neurons and oligodendrocytes. To identify each of these differentiated cell types, cell-specific markers can be used. These include GFAP (glial fibrillary acidic protein) for astrocytes, βIII-tubulin (part of the microtubular complex) for neurons, and O4 (oligodendrocyte surface antigen) and PLP (oligodendrocyte-specific transmembrane proteolipid) for oligodendrocytes (Fig. 1A).

To promote NS-5 cell differentiation we employed two strategies using two different types of differentiation media. Cells cultured in the first medium changed their morphology, finally generating astrocytes; the second medium promoted differentiation into a mixed culture of neurons and oligodendrocytes. To assess whether cell differentiation has an effect on *Disp3* expression, qRT-PCR was used to quantify and compare the levels of *Disp3* in each of the cell populations. This analysis revealed that *Disp3* levels are highest in undifferentiated cells and significantly decrease upon differentiation (Fig. 1B). The differentiation status of cells was verified by qRT-PCR using previously mentioned cell-type specific markers (Fig. S1). These results were further confirmed by immunofluorescence studies, which demonstrated reduced DISP3 protein expression when NS-5 cells differentiate (Fig. 1C). The status of cells in the various conditions was confirmed by immunofluorescence staining with specific markers. Staining of undifferentiated cells revealed either very low (βIII-tubulin) or undetectable (GFAP, O4) levels of neural cell-specific markers (not shown). To conclude, differentiation of NS-5 cells into astrocytes, neurons or oligodendrocytes results in reduced levels of both DISP3 mRNA and protein.

### Modulation of DISP3 expression in NS-5 cells

**Figure 2:**
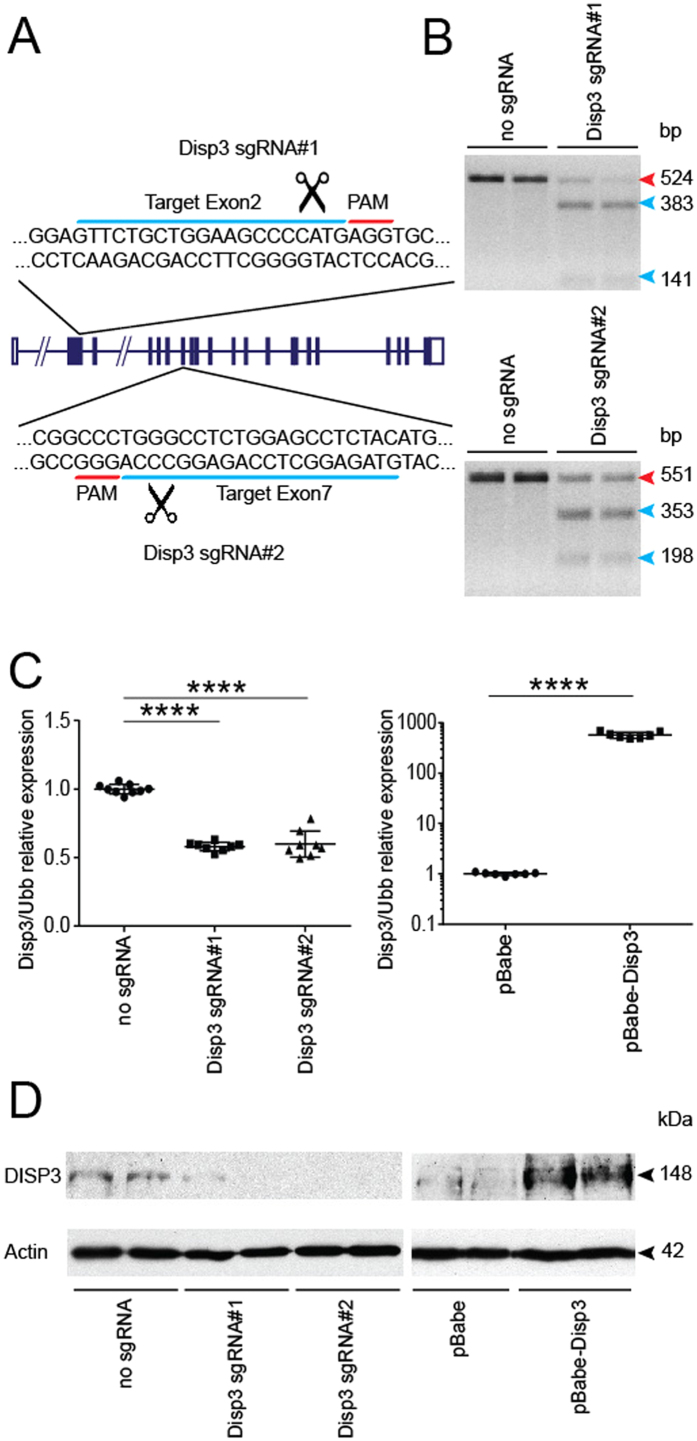
Modulation of DISP3 levels in NS-5 cells. (**A**) A scheme of the *Disp3* gene showing two sgRNA target sites for *Disp3* targeting via RNA-guided CRISPR/Cas9 endonuclease. The 20 nt target sequence (blue) is represented along with the neighboring PAM motif (red). (**B**) T7 endonuclease I assays confirm indels induced by Disp3 sgRNA#1 and Disp3 sgRNA#2 in two independent cell batches. The red arrow indicates the wild-type DNA fragment, blue arrows indicate mutant DNA fragments. (**C**) Quantitative RT-PCR analysis of *Disp3* mRNA. Cells infected with a lentivirus encoding Cas9 and Disp3 sgRNA#1 or Disp3 sgRNA#2 and control without sgRNA (left scatter plot); cells infected with a retrovirus encoding human *DISP3* (pBabe-Disp3) and control empty vector (pBabe) (right scatter plot). *Ubb* was used as a reference gene. Data represent the mean of biological replicates with error bars indicating standard deviation and the level of statistical significance (****P < 0.0001). Mean values: no sgRNA = 1.00, Disp3 sgRNA#1 = 0.58, Disp3 sgRNA#2 = 0.60, pBabe = 1.00, pBabe-Disp3 = 576. (**D**) Level of DISP3 protein confirmed by western blot. Actin served as a loading control.

To elucidate the effect of DISP3 expression on neural stem cell fate, the levels of *Disp3* expression were diminished using the CRISPR/Cas9 technology. Two custom designed single guide RNAs targeting exon 2 (Disp3 sgRNA#1) and exon 7 (Disp3 sgRNA#2) were used to impair expression of the *Disp3* gene (Fig. 2A). NS-5 cells were infected with a lentivirus containing Cas9 nuclease and either Disp3 sgRNA#1 or Disp3 sgRNA#2. After antibiotic selection, the presence of mutated alleles was confirmed using a batch T7 endonuclease I assay (Fig. 2B).

Additionally, NS-5 cells overexpressing DISP3 were generated by retroviral transduction with a DISP3-overexpression vector (pBabe-Disp3). The levels of *Disp3* mRNA in both novel NS-5 cell cultures (Disp3 sgRNAs and pBabe-Disp3) were determined by qRT-PCR (Fig. 2C) and later confirmed by Western blotting (Fig. 2D). Thus, using the CRISPR-Cas9 and retroviral transduction systems, we were able to efficiently modulate DISP3 expression in NS-5 cells.

### Modulated DISP3 expression in NS-5 cells modifies the phenotype of undifferentiated NSCs

**Figure 3:**
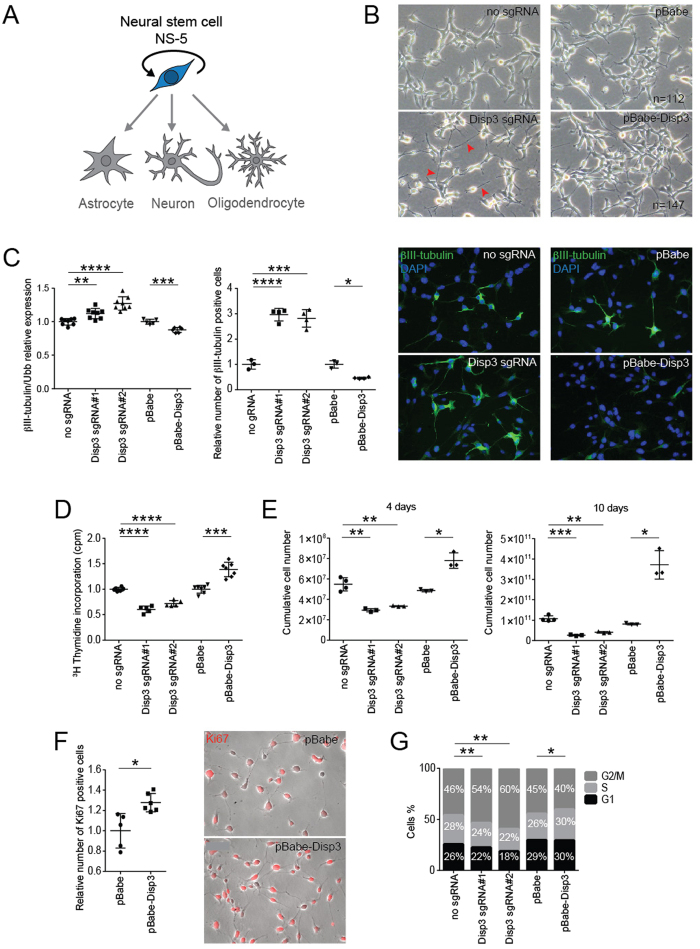
Modulation of DISP3 expression results in changes to undifferentiated NS-5 cells. (**A**) Under particular conditions, NS-5 cells are capable of self-renewal. (**B**) Modulation DISP3 expression leads to morphological changes in undifferentiated NS-5 cells. Representative phase-contrast images of Disp3 sgRNA, pBabe-Disp3 and relevant control cells are shown. Note the prolonged processes observed in Disp3 sgRNA cells (red arrows). (**C**) βIII-tubulin expression in Disp3 sgRNA, pBabe-Disp3 and control cells was quantified by qRT-PCR analysis (left scatter plot, *Ubb* was used as a reference gene) and by the Operetta High-Content Imagining System followed by Columbus software analysis (right scatter plot). According to the intensity of staining, cells were divided into three groups: cells with basal, medium and high intensity of βIII-tubulin staining. This analysis shows the fraction of βIII-tubulin-positive cells with the staining intensity exceeding the basal level and normalized to the number of total cells (nuclei). Representative immunofluorescent images of cells stained with βIII-tubulin (green) and DAPI (blue) are also shown. All data represent the mean of biological replicates with error bars indicating standard deviation and the level of statistical significance (*P < 0.05, **P < 0.01, ***P < 0.001, ****P < 0.0001). Mean values - left scatter plot: no sgRNA = 1.00, Disp3 sgRNA#1 = 1.12, Disp3 sgRNA#2 = 1.27, pBabe = 1.00, pBabe-Disp3 = 0.88; - right scatter plot: no sgRNA = 1.00, Disp3 sgRNA#1 = 2.97, Disp3 sgRNA#2 = 2.82, pBabe = 1.00, pBabe-Disp3 = 0.46. (**D**) Proliferation of Disp3 sgRNA, pBabe-Disp3 and control cells was measured by ^3^H-thymidine incorporation assays. All data represent the mean of biological replicates with error bars indicating standard deviation and the level of statistical significance (***P < 0.001, ****P < 0.0001). Mean values: no sgRNA = 1.00, Disp3 sgRNA#1 = 0.60, Disp3 sgRNA#2 = 0.72, pBabe = 1.00, pBabe-Disp3 = 1.39. (**E**) Disp3 sgRNA, pBabe-Disp3 and control cells were counted every other day. The growth rate was plotted as cumulative cell numbers. All data represent the mean of biological replicates with error bars indicating standard deviation and the level of statistical significance (*P < 0.05, **P < 0.01, ***P < 0.001). Mean values - left scatter plot (4 days): no sgRNA = 5.48 × 10^7^, Disp3 sgRNA#1 = 2.94 × 10^7^, Disp3 sgRNA#2 = 3.33 × 10^7^, pBabe = 4.86 × 10^7^, pBabe-Disp3 = 7.81 × 10^7^; - right scatter plot (10 days): no sgRNA = 1.07 × 10^11^, Disp3 sgRNA#1 = 0.27 × 10^11^, Disp3 sgRNA#2 = 0.41 × 10^11^, pBabe = 0.81 × 10^11^, pBabe-Disp3 = 3.72 × 10^11^. (**F**) Operetta High-Content Imaging System quantification of Ki-67 staining (left) and representative immunofluorescence images of pBabe-Disp3 and control cells stained with Ki-67 (red, right). All data represent the mean of biological replicates with error bars indicating standard deviation and the level of statistical significance (*P < 0.05). Mean values: pBabe = 1.00, pBabe-Disp3 = 1.28. (**G**) The effect of modified DISP3 expression on cell cycle progression was determined by flow cytometry analysis. The percentage of cells in the G1, S and G2/M phases of the cell cycle was counted and plotted. All data represent the mean of biological replicates, the level of statistical significance shows changes in the percentage of cells in the G2/M phase (*P < 0.05, **P < 0.01).

Previously, we have demonstrated that the process of NSC differentiation resulted in altered DISP3 expression. We next evaluated the possible effect of modulated DISP3 expression on undifferentiated cells maintained under self-renewal conditions (Fig. 3A).

Compared to control cells, both Disp3 sgRNAs and pBabe-Disp3 cell lines exhibited morphological changes upon cultivation in standard growth medium. The most evident change observed in Disp3 sgRNAs cells was formation of elongated cell processes, which indicated the onset of neuronal differentiation (Fig. 3B, left panels). Interestingly, cells overexpressing DISP3 (pBabe-Disp3) seemed to proliferate faster than the control cells. This phenomenon is illustrated in Fig. 3B, right panels, when after seeding a constant number of cells per dish and subsequent cultivation under the same conditions, there were more cells present in the pBabe-Disp3 culture compared to the pBabe control culture.

Moreover, the results of qRT-PCR and protein analysis demonstrated that in both Disp3 sgRNAs and pBabe-Disp3 cells, βIII-tubulin expression levels were altered (Fig. 3C). The same effect was observed using shRNA-induced knock-down in NS-5 cells (Fig. S2). Quantification of mRNA changes (Fig. 3C, left scatter plot) clearly illustrates that a decrease in DISP3 expression results in an increase in βIII-tubulin expression, whilst an increase in DISP3 levels causes the reverse effect. These results were confirmed by microscopy followed by automated image analysis of cells stained with a βIII-tubulin antibody. The analysis shows that compared to control cells, the number of βIII-tubulin-positive cells is higher in Disp3 sgRNA NS-5 cells and reduced in pBabe-Disp3 cells (Fig. 3C, right scatter plot). Representative images of this staining are shown in Fig. 3C, right panels. Moreover, in Disp3 sgRNA NS-5 cells, the fraction of highly and medium βIII-tubulin-stained cells is increased, whereas the percentage of cells with basal expression decreased (Fig. S3), which indicates that overall more cells have increased βIII-tubulin expression.

To elucidate the effect of DISP3 expression on maintaining the undifferentiated state of NS-5 cells, nestin (type-IV intermediate filament protein) mRNA levels were characterized by qRT-PCR. Analysis of both cell lines revealed changes in the nestin expression, which confirmed our previous data suggesting that reduction of DISP3 expression promotes spontaneous differentiation of NS-5 cells, whilst its overexpression helps to maintain the cell stemness (Fig. S4).

To quantify cell proliferation in Disp3 sgRNAs and pBabe-Disp3 cells, a thymidine incorporation assay was performed. The proliferation rate of NS-5 cells overexpressing DISP3 was increased compared to control cells, whilst the proliferation rate of Disp3 sgRNAs was reduced (Fig. 3D, Fig. S5). The growth rate of Disp3 sgRNAs and pBabe-Disp3 cells was quantified every other day and plotted as cumulative cell numbers. Figure 3E shows two selected days from the growth curve (Fig. S6) and corroborates that DISP3 overexpression provides a growth advantage. These results were confirmed using Ki-67 staining (marker of cell proliferation). Compared to control cells, pBabe-Disp3 cells displayed a significantly higher number of Ki-67-positive stained cells (Fig. 3F). Finally, to determine the effect of DISP3 expression on the cell cycle, flow cytometry analysis was performed. This analysis revealed that compared to control cells, both disruption and overexpression of the *Disp3* gene leads to an altered distribution of cells in distinct phases of the cell cycle (Fig. 3G).

### Changes in DISP3 expression affect NS-5 cell fate decisions

**Figure 4:**
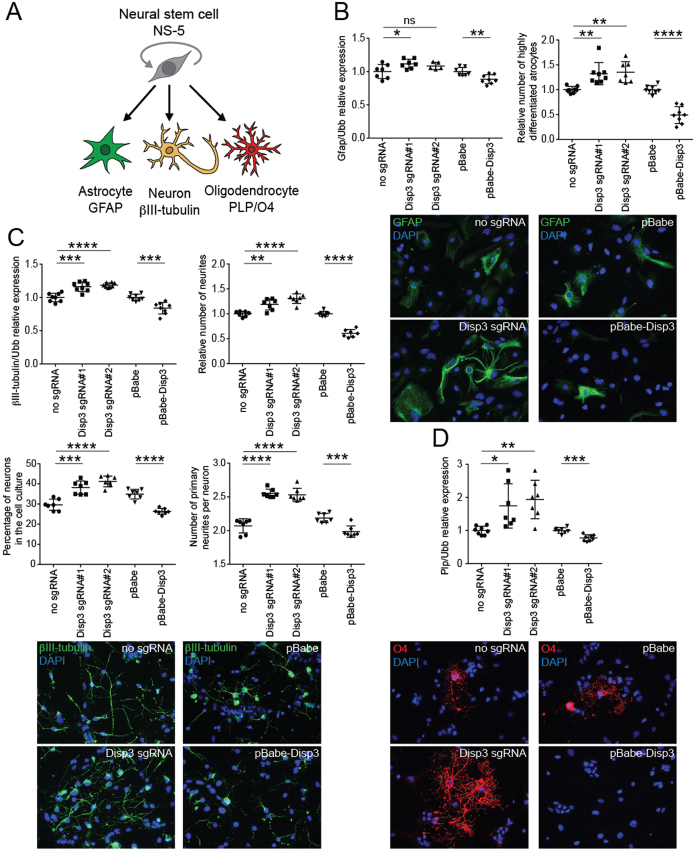
Changes in DISP3 expression levels affect NS-5 differentiation patterns. (**A**) Multipotent neural stem cells NS-5 give rise to astrocytes, neurons and oligodendrocytes. Cell type-specific markers GFAP (astrocytes), βIII-tubulin (neurons) and O4 (oligodendrocytes) were analyzed. (**B**) qRT-PCR analysis of *Gfap* mRNA expression (left scatter plot, *Ubb* was used as a reference gene) and GFAP staining of cells after astrocyte differentiation analyzed by the Operetta High-Content Imagining System followed by Columbus software analysis (right scatter plot). This analysis shows the fraction of highly differentiated astrocytes, which were recognized and quantified based on their shape, size, and intensity of staining and normalized to the number of total cells (nuclei). Representative immunofluorescent images of cells stained with GFAP (green) are shown. All data represent the mean of biological replicates with error bars indicating standard deviation and the level of statistical significance (*P < 0.05, **P < 0.01, ****P < 0.0001). Mean values - left scatter plot: no sgRNA = 1.00, Disp3 sgRNA#1 = 1.12, Disp3 sgRNA#2 = 1.08, pBabe = 1.00, pBabe-Disp3 = 0.88; - right scatter plot: no sgRNA = 1.00, Disp3 sgRNA#1 = 1.32, Disp3 sgRNA#2 = 1.35, pBabe = 1.00, pBabe-Disp3 = 0.49. (**C**) qRT-PCR analysis of βIII-tubulin mRNA expression (upper left scatter plot, *Ubb* was used as a reference gene) and the numbers of βIII-tubulin-stained neurites (upper right scatter plot) after neuronal differentiation counted by the Operetta High-Content Imagining System followed by Columbus software analysis using the neurite finding tool. The analysis shows the relative number of neurites normalized to the number of total cells (nuclei). The lower left scatter plot displays the percentage of neurons in each cell culture analyzed manually with ImageJ. The lower right scatter plot shows the number of primary neurites recalculated per neuron. All data represent the mean of biological replicates with error bars indicating standard deviation and the level of statistical significance (**P < 0.01, ***P < 0.001, ****P < 0.0001). Representative immunofluorescent images of cells stained with βIII-tubulin (green) are shown at the bottom. Mean values - upper left scatter plot: no sgRNA = 1.00, Disp3 sgRNA#1 = 1.16, Disp3 sgRNA#2 = 1.19, pBabe = 1.00, pBabe-Disp3 = 0.84; - upper right scatter plot: no sgRNA = 1.00, Disp3 sgRNA#1 = 1.19, Disp3 sgRNA#2 = 1.31, pBabe = 1.00, pBabe-Disp3 = 0.61; - lower left scatter plot: no sgRNA = 29.56, Disp3 sgRNA#1 = 38.13, Disp3 sgRNA#2 = 41.19, pBabe = 34.91, pBabe-Disp3 = 26.33; - lower right scatter plot: no sgRNA = 2.07, Disp3 sgRNA#1 = 2.55, Disp3 sgRNA#2 = 2.53, pBabe = 2.18, pBabe-Disp3 = 1.99. (**D**) qRT-PCR analysis of *Plp* mRNA expression after oligodendrocyte differentiation. *Ubb* was used as a reference gene. Representative immunofluorescent images of cells stained with O4 (red) are shown. All data represent the mean of biological replicates with error bars indicating standard deviation and the level of statistical significance (*P < 0.05, **P < 0.01, ***P < 0.001). Mean values: no sgRNA = 1.00, Disp3 sgRNA#1 = 1.74, Disp3 sgRNA#2 = 1.93, pBabe = 1.00, pBabe-Disp3 = 0.78.

Given that NS-5 cells can be driven to differentiate into neural cell types *in vitro*, we wondered whether modifying DISP3 expression would affect the NSC differentiation fates. Cells either under- or over-expressing DISP3 were analyzed to determine whether the expression levels of neural-specific markers were altered in NS-5 cells incubated under specific conditions to promote differentiation into either astrocytes, neurons or oligodendrocytes (Fig. 4A).

GFAP is a type-III intermediate filament protein that has previously been used to identify astrocytes. During astrocyte differentiation, the level of *Gfap* mRNA was significantly increased from undetectable on day 0 to relatively high in fully differentiated control NS-5 cells (Fig. S1). GFAP expression in Disp3 sgRNAs and pBabe-Disp3 astrocytes followed a similar pattern to control cells; however, at the end of differentiation the increased *Gfap* mRNA levels in Disp3 sgRNAs and the reduced *Gfap* mRNA levels in pBabe-Disp3 cells indicate an impact of DISP3 on the astrocyte differentiation (Fig. 4B left scatter plot, Fig. S7). Whilst *Gfap* mRNA expression did not differ greatly among the various cell types, there was a definite impact at the protein level. Cells stained with antibodies against GFAP were recognized and quantified based on their shape, size, and intensity of staining and the results revealed that compared to control cells, Disp3 sgRNAs cells in culture had an increased number of highly differentiated astrocytes. The reverse effect was observed in pBabe-Disp3 cells (Fig. 4B right scatter plot, representative images).

To examine neuronal differentiation in NS-5 cells, βIII-tubulin mRNA levels were quantified. Increased βIII-tubulin mRNA levels were observed in Disp3 sgRNA cells whilst pBabe-Disp3 cells showed a reduction (Fig. 4C upper left scatter plot, Fig. S7). This result demonstrates that modulation of DISP3 expression affects βIII-tubulin mRNA levels under neuronal differentiation conditions in the same direction as under the growth/proliferation conditions (Fig. S8). Analysis of the morphology of control NS-5-derived neurons revealed long prototypical neuronal projections (neurites), which expressed high levels of βIII-tubulin protein. The number of neurites normalized to the total cell number has been selected as an initial readout for neuronal differentiation.

The numbers of neurites derived from each NS-5 cell culture were automatically quantified and revealed that, compared to the control culture, there were more neurites per cell in Disp3 sgRNAs and less neurites in pBabe-Disp3 cell culture (Fig. 4C upper right scatter plot). Evaluation of the number of neurons in differentiated neuronal culture is difficult using the automated image analysis because of their dense cytoplasm and thin processes. Nevertheless, we were able to analyze the number of neurons manually using the ImageJ software. In differentiated culture, only βIII-tubulin-positive cells with extending neurites and dense cytoplasm were considered as neurons. The analysis shows that compared to control cells, the percentage of fully differentiated neurons is higher in Disp3 sgRNA NS-5 cell culture and reduced in pBabe-Disp3 cells (Fig. 4C lower left scatter plot). Moreover, ImageJ analysis of the absolute number of primary neurites recalculated per neuron in each NS-5 cell culture showed that, compared to the control culture, there were more primary neurites in Disp3 sgRNAs and less primary neurites in pBabe-Disp3 neurons (Fig. 4C lower right scatter plot, bottom representative images).

Finally, the effect of DISP3 expression on oligodendrocyte differentiation was determined by quantitating the level of *Plp* mRNA and O4 protein expression in NS-5 cell cultures after differentiation. PLP is an oligodendrocyte-specific transmembrane proteolipid protein that is required for neuronal myelination. O4 is an antigen located on the surface of oligodendrocytes. *Plp* mRNA levels increased in Disp3 sgRNAs cells while decreased in pBabe-Disp3 cells. Similar findings were obtained when cells were stained with the O4 antibody (Fig. 4D, Fig. S7). Unfortunately, due to the small fraction of cells positive for oligodendrocyte markers (especially in pBabe-Disp3 cells), we were unable to quantify this staining and therefore, show only a representative picture.

In summary, modulation of DISP3 expression affects NS-5 differentiation. Compared to control cell cultures, Disp3 sgRNAs cells generated more highly differentiated astrocytes and showed increased expression of *Plp* mRNA, which is specific for oligodendrocytes. Similarly, the expression of neuron-specific βIII-tubulin mRNA was increased, and as such, these cells exhibited increased numbers of neurites. Furthermore, compared to control cells, the number of fully differentiated neurons was higher in Disp3 sgRNA NS-5 cell culture and these neurons had increased numbers of primary neurites. In pBabe-Disp3 cells, the differentiation potential was much lower regardless of the differentiation strategy used.

### Modified levels of DISP3 lead to altered gene expression in NS-5 cells

**Figure 5:**
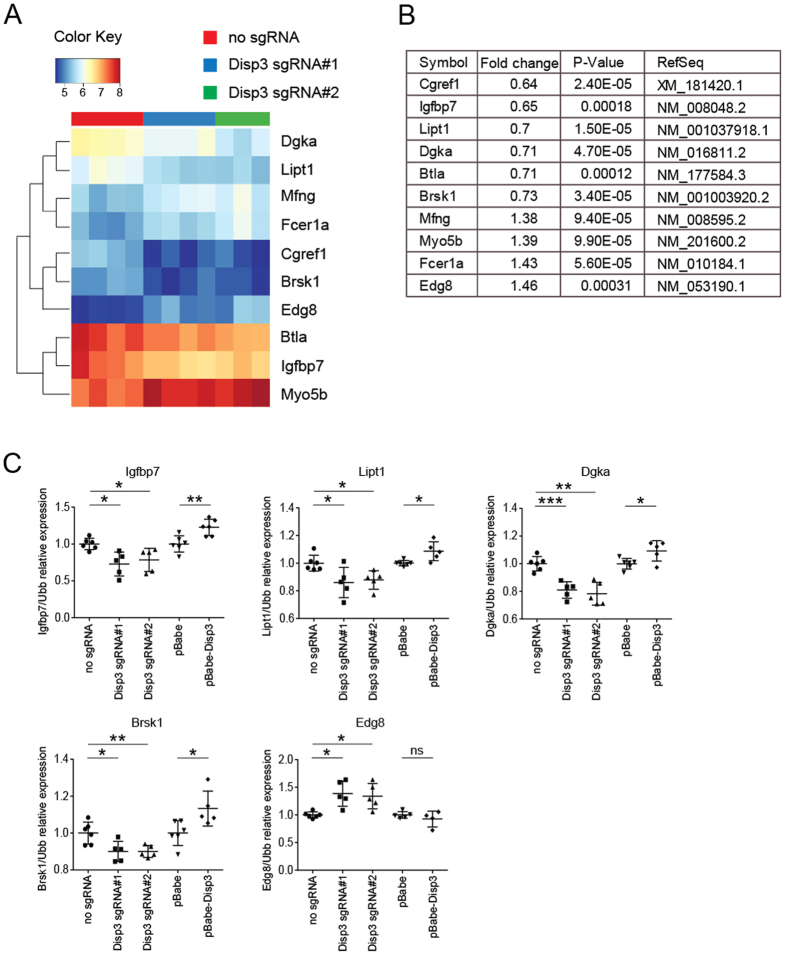
*Disp3* gene expression modulation alters gene expression in NS-5 cells. A heatmap (**A**) and a list of genes (**B**) with altered expression in Disp3 sgRNA cells relative to control cells was obtained by microarray expression profiling. (**C**) Relative expression of identified genes in Disp3 sgRNA, pBabe-Disp3 and control cells was confirmed by an independent qRT-PCR experiment. *Ubb* was used as a reference gene. Data represent the mean of biological replicates with error bars indicating standard deviation and the level of statistical significance (*P < 0.05, **P < 0.01, ***P < 0.001). Mean values - Igfbp7: no sgRNA = 1.00, Disp3 sgRNA#1 = 0.73, Disp3 sgRNA#2 = 0.78, pBabe = 1.00, pBabe-Disp3 = 1.23; - Lipt1: no sgRNA = 1.00, Disp3 sgRNA#1 = 0.86, Disp3 sgRNA#2 = 0.87, pBabe = 1.00, pBabe-Disp3 = 1.09; - Dgka: no sgRNA = 1.00, Disp3 sgRNA#1 = 0.81, Disp3 sgRNA#2 = 0.78, pBabe = 1.00, pBabe-Disp3 = 1.09; - Brsk1: no sgRNA = 1.00, Disp3 sgRNA#1 = 0.90, Disp3 sgRNA#2 = 0.90, pBabe = 1.00, pBabe-Disp3 = 1.13; - Edg8: no sgRNA = 1.00, Disp3 sgRNA#1 = 1.39, Disp3 sgRNA#2 = 1.34, pBabe = 1.00, pBabe-Disp3 = 0.93.

To identify genes in NS-5 cells affected by altered DISP3 expression, we performed microarray analysis. Total RNAs isolated from Disp3 sgRNA#1, Disp3 sgRNA#2 and control cells were hybridized to the Illumina Mouse Expression BeadChips. On the basis of the obtained expression profiles we established a heatmap (Fig. 5A) and a compiled list of common genes (Fig. 5B) with significantly altered expression in both Disp3 sgRNA cultures. The expression levels of the identified genes were confirmed by qRT-PCR in independent Disp3 sgRNA, pBabe-Disp3 and control cell cultures. Our results show that modified expression levels of DISP3 lead to altered mRNA levels of insulin-like growth factor binding protein 7 (*Igfbp7*), lipoyltransferase 1 (*Lipt1*), diacylglycerol kinase alpha (*Dgka*), brain serine/threonine kinase 1 (*Brsk1*) and endothelial differentiation sphingolipid G-protein-coupled receptor 8 (*Edg8*) (Fig. 5C). Interestingly, all of these identified genes were described to function either in neurodevelopment and/or have been implicated in various tumors.

## Discussion

In this study we examined the role of DISP3 in NSC proliferation and differentiation. We have demonstrated in NS-5 cells that the levels of DISP3 expression significantly decrease upon differentiation. We performed both loss-of-function and gain-of-function experiments to determine the effect of DISP3 on the NSC phenotype and differentiation potential. When NSCs were maintained under growth/proliferation conditions, both Disp3 sgRNAs and pBabe-Disp3 cells underwent significant morphological changes. The most obvious alteration was observed in Disp3 sgRNAs cells. Even though the mRNA expression of βIII-tubulin did not much differ from control cells, the number of βIII-tubulin-positive cells detected by immunofluorescence staining and automated counting was increased nearly three times. The analysis also revealed that not only the fraction of βIII-tubulin-positive cells was higher in Disp3 sgRNA NS-5 cell culture, but also that the number of highly stained cells was significantly increased. The observed high expression levels of NSC marker nestin in Disp3 sgRNA NS-5 cells under the growth/proliferation conditions, which were only slightly lower than in control cells, demonstrate the undifferentiated character of these cells. Nevertheless, the increased proportion of βIII-tubulin-positive cells indicates the onset of neuronal differentiation. Interestingly, cells overexpressing DISP3 demonstrated a significantly faster proliferation rate than control cells.

In the postnatal cerebellum, the expression of DISP3 is controlled by ATOH1, a transcription factor that plays a major role in cell proliferation, cell migration and initiation of the cell differentiation program^34^. Interestingly, ATOH1 is also implicated in promoting medulloblastoma formation^35^. Analysis of Oncomine datasets revealed that *DISP3* expression is significantly elevated in human medulloblastoma^21^. Medulloblastoma is the most common malignant pediatric brain tumor and can be classified into at least four distinct molecular subgroups that vary according to clinical signs and molecular markers. The most aggressive forms of the disease are the non-SHH/WNT subtypes^36^. Each of these subtypes is characterized by expression of markers that are found in embryonic or adult stem cells, as well as in neural “cancer stem cells” (CSC). It has been hypothesized that sustained tumor growth is restricted to CSCs, which display many similarities to NSCs in their capacity to self-renew and differentiate^37^. Neural CSCs are able to grow in serum-free media containing EGF and FGF, the two growth factors that promote its “dedifferentiated” status^8^,^38^,^39^.

Immunohistochemical evidence suggests that particular medulloblastoma molecular subgroups arise from distinct cellular origins. WNT-subtype tumors infiltrate the dorsal brainstem, whereas SHH medulloblastomas originate from external germinal layer progenitor (EGL) cells^40^. The origins of non-SHH/WNT subtypes 3 and 4 remain to be defined; nevertheless, it has been demonstrated that the master regulator transcription factors of subgroup 4 are active in neurons that originate from early progenitors of the upper rhombic lip^41^. In our previous paper, we used the multipotent cerebellar progenitor cell line C17.2, which was generated from isolated cerebellar neural cells from the EGL layer. The study demonstrated that ectopic overexpression of DISP3 promotes cell proliferation, alters tumorigenic gene expression and modifies the differentiation profile of DISP3-expressing C17.2 cells^21^.

To elucidate whether modulation of DISP3 expression has any effect on the NSC differentiation potential, we determined the expression levels of neural-specific markers in differentiated Disp3 sgRNA cells, pBabe-Disp3 and control cells using both manual and automated microscopy methods. Compared to control cell cultures, differentiated Disp3 sgRNAs cells yielded a greater proportion of neurons and highly differentiated astrocytes. Analysis of Disp3 sgRNA cell culture also showed a higher number of fully differentiated neurons with more primary neurites compared to control cells. In contrast, pBabe-Disp3 cells demonstrated impaired differentiation.

DISP3 is an SSD-containing protein with a phylogenetic relationship to other members of this family. Previously identified genetic mutations within SSD-containing protein family members have revealed NSC self-renewal and differentiation defects. Mutations in the *NPC1* gene cause the Niemann-Pick type C1 lysosomal lipid storage disorder that is characterized by multisystem defects including demyelination and progressive neurodegeneration^42^. *Npc1* mutations lead to reduced NSC self-renewal, impaired neurosphere generation and altered astrocyte morphology^43^. Current research indicates that an imbalance between NSC proliferation and differentiation is the major cause of many neurodevelopmental disorders. To date, research of developmental disabilities was mostly based on gene mutation studies in case/control cohorts. However, induced pluripotent stem cells (iPSCs) generated from affected patients are now being used to measure the extent of the neural proliferation/differentiation deficit, which may, in many cases, correspond with the degree of impairment. This appears to be critical for formulating appropriate treatment strategies, as these neurodevelopmental disorders are considered as multi-cause, complex diseases of NSC proliferation and differentiation rather than as a cluster of particularly impaired molecular pathways^44^. Upon neuronal differentiation, human iPSCs containing the most frequent *NPC1* mutation exhibit premature cell death and dysfunction of important pathways, including calcium and WNT signaling^45^. Another neurological disease involving altered NSC function is the Smith-Lemli-Opitz syndrome (SLOS). SLOS is a cholesterol synthesis disorder caused by a mutation within the *DHCR7* gene. Impaired DHCR7 activity results in lower cholesterol biosynthesis and higher 7-dehydrocholesterol levels^46^. The altered sterol composition within cells then leads to central nervous system malformations, as well as cognitive and behavioral abnormalities^47^,^48^. SLOS-derived iPSCs exhibit accelerated neuronal differentiation, formation of long neuronal projections and increased expression of neuronal markers^49^. Importantly, *DISP3* was identified among the top differentially expressed transcripts in neurons derived from *TSC2*-deleted pluripotent stem cells. Mutations in either *TSC1* or *TSC2* have been determined to cause tuberous sclerosis, a severe multisystem disorder presented by neuropsychiatric symptoms including autism, intellectual disability, and epilepsy^50^. *TSC2* deletion alters neuronal proliferation and differentiation and leads to altered neuronal synaptogenesis and transmission paralleled by molecular changes in pathways associated with autism^51^.

To support the observed phenotypes, we conducted gene expression profiling of NS-5 cells with disrupted DISP3 expression. The analysis revealed a number of genes with altered mRNA levels. These included *Igfbp7, Lipt1, Dgka, Brsk1* and *Edg8*, which have known neurodevelopmental functions and/or have been implicated in various tumors. Both neurodevelopment and oncogenesis are multi-step processes in which altered signal transduction pathway activation can have pleiotropic effects depending on the specific gene expression profile in the particular cell type and during the specific time-window, and recent findings show that many genes are implicated in both processes^52^,^53^.

IGFBP7 belongs to the superfamily of insulin-like growth factor (IGF)-binding proteins (IGFBPs), which have been described to function in tumorigenesis in a tissue type- and tumor pathology-dependent manner^54^,^55^,^56^,^57^,^58^,^59^. In glioblastoma, IGFBP7 is upregulated and its expression level within the tumor correlates with its histological grade and patient prognosis^60^. In a noncarcinogenic brain, it was shown that IGFBP7 downregulation resulted in increased hippocampal neuron survival^61^. Interestingly, brain tissue and blood serum isolated from Alzheimer’s disease patients demonstrated IGFBP7 upregulation^62^. Another gene whose expression was downregulated in Disp3-sgRNA cells was *Dgka*. DGKA is a member of the diacylglycerol kinase family and is expressed specifically within oligodendrocytes of the CNS. It has also been proposed to function in myelin formation and metabolism^63^ and was identified as a marker for a depressive disorder relapse^64^. Many studies have also suggested a role for DGKA in tumorigenesis^65^,^66^,^67^. Interestingly, attenuation of DGKA activity in glioblastoma cells induced apoptosis^68^,^69^. BRSK1 is an AMP-activated serine/threonine kinase, which is specifically expressed in the brain and is known to play an essential role in neuronal polarization. It has been reported that the loss of both BRSK1 and its related protein BRSK2 is required to affect neuronal differentiation. Such double-knockout mice show a smaller forebrain and an abnormally thin cortex with disordered segregation of particular neuronal subtypes. Double-mutant neurons displayed changes in morphology and axons that were difficult to distinguish from dendrites^70^. BRSK1 also plays an important role in the regulation of cell cycle progression through controlling centrosome duplication via phosphorylation of γ-tubulin^71^. The only gene whose expression was upregulated in Disp3-sgRNA cells was *Edg8*. This gene encodes a sphingosine 1-phosphate G-protein-coupled receptor that is expressed predominantly in oligodendrocytes and astrocytes^72^. In the developing brain, EDG8 is expressed in radial glial cells and is involved in transforming these cells into astrocytes^73^. In the course of oligodendrocytes differentiation, EDG8 is expressed in both immature and mature stages of myelin-forming cell and promotes survival of mature oligodendrocytes^74^. Overall, the functions of the identified DISP3-sensitive genes correlate well with our experimental results that demonstrate that DISP3 expression levels impact NSC proliferation and differentiation.

In this study, we utilized the mouse neural stem cell line NS-5, which is capable of differentiating into neurons, astrocytes and oligodendrocytes. Currently, this is one of the few *in vitro* model systems available to investigate the effect of DISP3 downregulation on NSC self-renewal and differentiation. We used the CRISPR-Cas9 technology to reduce the endogenously high levels DISP3 in NS-5 cells in order to assess the ability of these transgenic cells to self-renew and differentiate. NSC proliferation and differentiation are tightly controlled processes that require subtle spatial and temporal adjustments; otherwise, NSCs that differentiate either prematurely or late will ultimately affect the overall number of neural cells generated. Taken together, our findings demonstrate that DISP3 may be one of the players that regulate the transition of NSC from self-renewal to differentiation.

## Additional Information

**How to cite this article:** Konířová, J. *et al*. Modulated DISP3/PTCHD2 expression influences neural stem cell fate decisions. *Sci. Rep.* **7**, 41597; doi: 10.1038/srep41597 (2017).

**Publisher's note:** Springer Nature remains neutral with regard to jurisdictional claims in published maps and institutional affiliations.

## Supplementary Material

Supplementary Information
