# Supplementary material for: Modulated DISP3/PTCHD2 expression influences neural stem cell fate decisions
Source: Sci Rep. 2017 Jan 30;7:41597. doi: 10.1038/srep41597 (PMC5278513; doi:10.1038/srep41597)
Supplement: Supplementary Information [file srep41597-s1.pdf]

Supplementary information

Modulated DISP3/PTCHD2 expression influences neural stem cell fate decisions

Jana Konířová, Jana Oltová, Alicia Corlett, Justyna Kopycińska, Michal Kolář, Petr Bartůněk* and Martina Zíková*

Institute of Molecular Genetics AS CR v.v.i., Vídeňská 1083, 142 20 Prague 4, Czech Republic

*Corresponding author:

Petr Bartůněk
Institute of Molecular Genetics AS CR v.v.i.
Vídeňská 1083, 142 20 Prague 4, Czech Republic
Tel. +420-241063117
Email: bartunek@img.cas.cz

Martina Zíková
Institute of Molecular Genetics AS CR v.v.i.
Vídeňská 1083, 142 20 Prague 4, Czech Republic
Tel. +420-241063113
Email: mzikova@img.cas.cz

Supplementary information

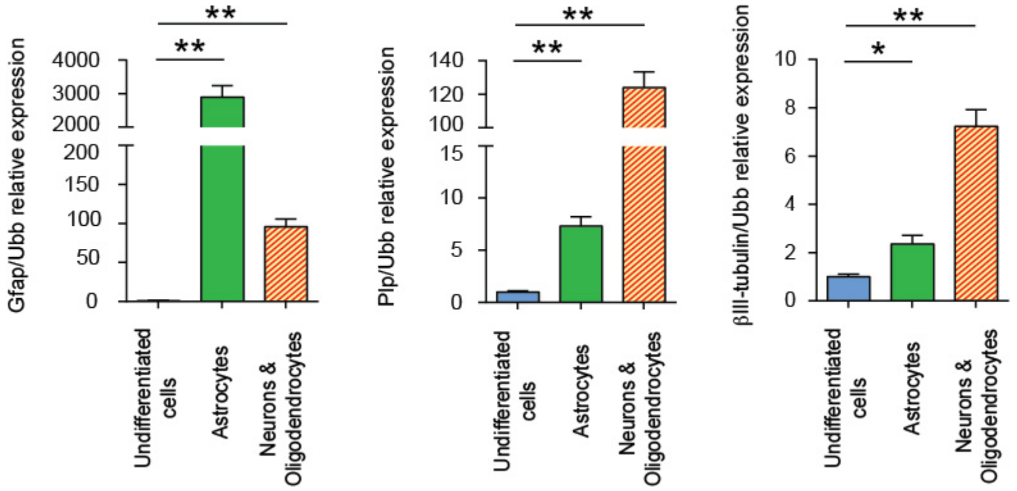

Supplementary Figure 1: Quantitative RT-PCR analysis of *Gfap, Plp* and βIII-tubulin mRNA expression in undifferentiated and differentiated NS-5 cells. *Ubb* was used as a reference gene. Bars represent the mean of three independent samples with error bars indicating standard deviation and the level of statistical significance (*P< 0.05, **P< 0.01). Mean values - Gfap relative expression: undifferentiated cells=1.00, astrocytes=2900, neurons and oligodendrocytes=96; - Plp relative expression: undifferentiated cells=1.00, astrocytes=7.30, neurons and oligodendrocytes=124; - βIII-tubulin relative expression: undifferentiated cells=1.00, astrocytes=2.36, neurons and oligodendrocytes=7.23.

Supplementary information

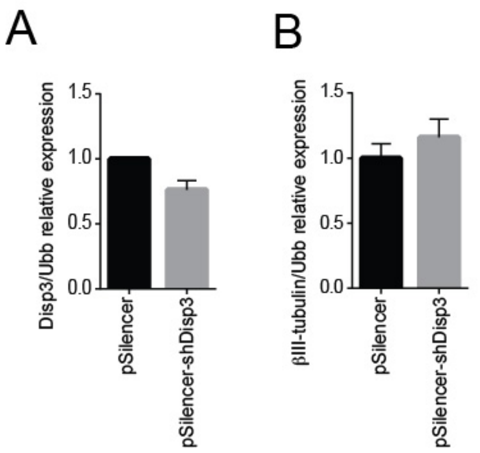

Supplementary Figure 2: Downregulation of DISP3 by shRNA leads to altered expression levels of βIII-tubulin.

(A) qRT-PCR analysis of *Disp3* expression in control (pSilencer) and shDisp3-treated NS-5 cells. *Ubb* was used as a reference gene. Bars represent the mean of three biological replicates with error bars indicating standard deviation. Mean values: pSilencer=1.00, pSilencer-shDisp3=0.76.

(B) qRT-PCR analysis of βIII-tubulin mRNA expression in control (pSilencer) and shDisp3-treated NS-5 cells. *Ubb* was used as a reference gene. Bars represent the mean of three biological replicates with error bars indicating standard deviation. Mean values: pSilencer=1.00, pSilencer-shDisp3=1.16.

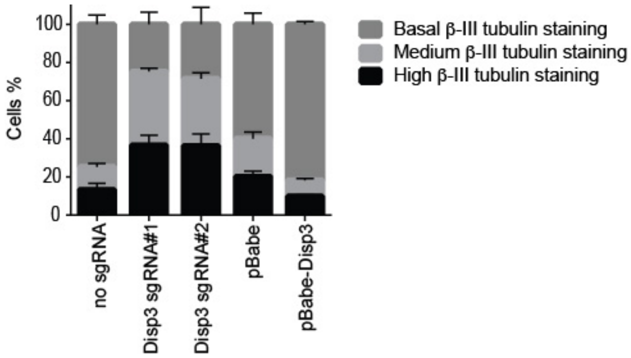

Supplementary Figure 3: Distribution of undifferentiated NS-5 cells based on β-III tubulin staining.

βIII-tubulin staining of undifferentiated NS-5 cells quantified by the Operetta High-Content Imagining System followed by Columbus software analysis. According to the intensity of βIII-tubulin staining, cells were divided into three groups: basal, medium and high intensity of staining. Data represent the percentage of cells in each particular group and the mean of biological replicates with error bars indicating standard deviation. Mean values - high βIII-tubulin staining: no sgRNA=13.47, Disp3 sgRNA#1=36.76, Disp3 sgRNA#2=36.40, pBabe=20.29, pBabe-Disp3=9.91; - medium βIII-tubulin staining: no sgRNA=11.92, Disp3 sgRNA#1=38.56, Disp3 sgRNA#2=35.18, pBabe=19.93, pBabe-Disp3=8.50; - basal βIII-tubulin staining: no sgRNA=74.61, Disp3 sgRNA#1=24.68, Disp3 sgRNA#2=28.42, pBabe=59.78, pBabe-Disp3=81.59.

Supplementary information

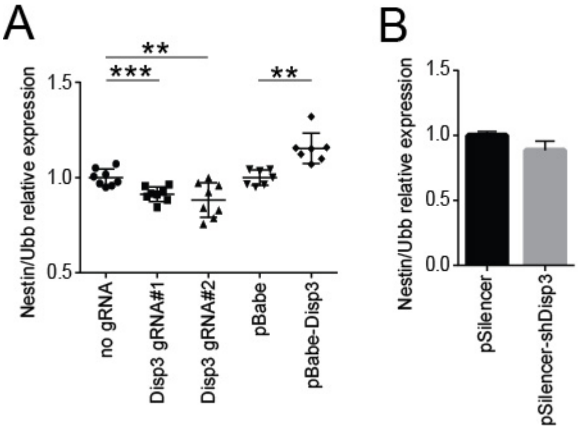

Supplementary Figure 4: Altered expression of nestin in Disp3 sgRNA (pSilencer-shDisp3) and pBabe-Disp3 cells.

(A) qRT-PCR analysis of nestin mRNA expression in Disp3 sgRNA, pBabe-Disp3 and control cells. *Ubb* was used as a reference gene. Data represent the mean of biological replicates with error bars indicating standard deviation and the level of statistical significance (**P< 0.01, ***P< 0.001). Mean values: no sgRNA=1.00, Disp3 sgRNA#1=0.91, Disp3 sgRNA#2=0.88, pBabe=1.00, pBabe-Disp3=1.15.

(B) qRT-PCR analysis of nestin mRNA expression in control (pSilencer) and shDisp3-treated NS-5 cells. *Ubb* was used as a reference gene. Data represent the mean of three biological replicates with error bars indicating standard deviation. Mean values: pSilencer=1, pSilencer-shDisp3=0.88.

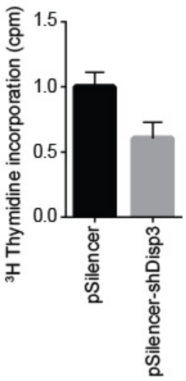

Supplementary Figure 5: Downregulation of DISP3 expression levels by shRNA leads to reduced NS-5 cell proliferation.

Proliferation of control (pSilencer) and shDisp3-treated NS-5 cells was measured by a ^{3}H-thymidine incorporation assay. Bars represent the mean of three biological replicates with error bars indicating standard deviation. Mean values: pSilencer=1.00, pSilencer-shDisp3=0.60.

Supplementary information

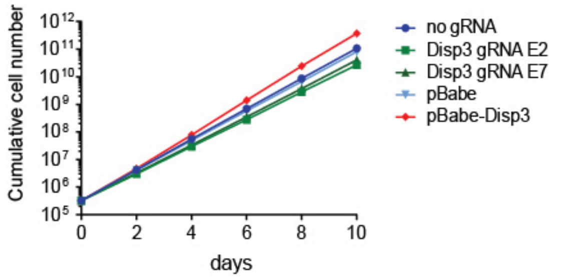

Supplementary Figure 6: Cell proliferation is altered in Disp3 sgRNA and pBabe-Disp3 NS-5 cells. Disp3 sgRNA, pBabe-Disp3 and control cells were counted every other day. The growth rate was plotted as cumulative cell numbers.

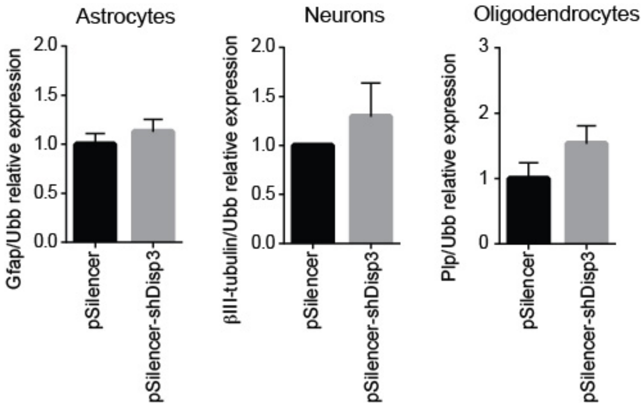

Supplementary Figure 7: Downregulation of DISP3 expression by shRNA promotes differentiation of NS-5 cells.

qRT-PCR analysis of *Gfap*, βIII-tubulin and *Plp* mRNA expression in control (pSilencer) and shDisp3 cells at days 4 (astrocytes) and 9 (neurons and oligodendrocytes) of differentiation. *Ubb* was used as a reference gene. Bars represent the mean of three biological replicates with error bars indicating standard deviation. Mean values - Gfap relative expression: pSilencer=1.00, pSilencer-shDisp3=1.13; - βIII-tubulin relative expression: pSilencer=1.00, pSilencer-shDisp3=1.30; - Plp relative expression: pSilencer=1.00, pSilencer-shDisp3=1.54.

Supplementary information

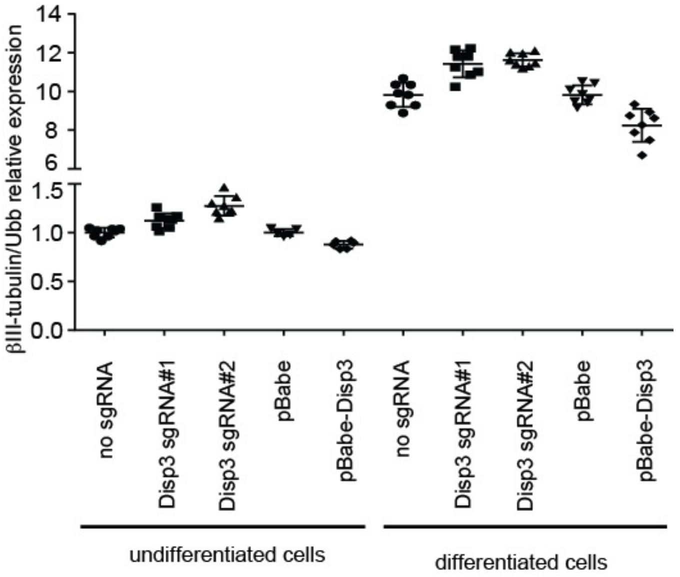

Supplementary Figure 8: Changes in DISP3 expression levels affect βIII-tubulin expression under both growth/proliferation and differentiation conditions. βIII-tubulin expression was quantified by qRT-PCR analysis (*Ubb* was used as a reference gene). Data represent the mean of biological replicates with error bars indicating standard deviation. Mean values - undifferentiated cells: no sgRNA=1.00, Disp3 sgRNA#1=1.12, Disp3 sgRNA#2=1.27, pBabe=1.00, pBabe-Disp3=0.88; - differentiated cells: no sgRNA=9.82, Disp3 sgRNA#1=11.42, Disp3 sgRNA#2=11.63, pBabe=9.82, pBabe-Disp3=8.24.
